# Physicochemical properties of Kakol (*Suaeda aegyptiaca*) essential oil nanoemulsion and its effect on the storage quality of rainbow trout (*Oncorhynchus mykiss*) during cold storage

**DOI:** 10.1002/fsn3.3480

**Published:** 2023-06-15

**Authors:** Payam Zibaee, Mohammad‐Amin Shamekhi

**Affiliations:** ^1^ Department of Food Science and Technology, Sarvestan Branch Islamic Azad University Sarvestan Iran

**Keywords:** bioactivity, minced rainbow trout fish, nanoemulsion, shelf‐life, *Suaeda aegyptiaca*

## Abstract

The study aims to analyze the chemical composition of *Suaeda aegyptiaca* essential oil (PSAE) by GC–MS, produce the nanoemulsified essential oil (NSAE) using ultrasound, and compare the antimicrobial and antioxidant activity of the PSAE and NSAE in laboratory medium and rainbow trout fish (*Oncorhynchus mykiss*). Geranyl‐acetone (30.52%) and p‐Vinylguaiacol (10.66%), and (e)‐β‐ionone (7.79%) were the main PSAE chemical compounds. The mean droplet size diameter, polydispersity index, and viscosity of NSAE were 179.67 nm, 0.255, and 0.96 cP, respectively. PSAE and NSAE showed a moderate antiradical potential against DPPH‐ and ABTS‐free radicals (50 < IC_50_ < 250 μg mL^−1^). There was no significant difference between antiradical scavenging of PSAE and NSAE (*p* > .05). *E. faecalis* and *K. pneumonia* were the most and lowest sensitive bacteria to PSAE and NSAE, respectively. Examining different treatments on the shelf‐life of minced fish showed that Kakol essential oil could improve the shelf‐life of fish between 12.5% and 60% (depending on quality index). There was no significant difference between the bioactivity of PSAE and NSAE, which means that the nanoemulsion showed acceptable performance at lower essential oil concentrations.

## Graphical abstract

Chemical analysis of Kakol (*Suaeda aegyptiaca*) essential oil. Physical stability of the nanoemulsified Kakol essential oil. The Moderate antioxidant potential of Kakol essential oil (pure and emulsified). Comparable antimicrobial activity of Kakol essential oil with tetracycline. Improving the shelf‐life of rainbow trout fish with Kakol essential oil.

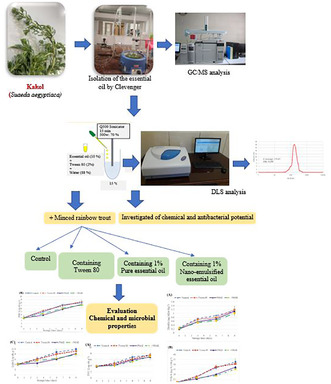

## 1 INTRODUCTION

By increasing public awareness about the side effects of chemical preservatives, consumer demand and scientific interest in the use of natural food additives is increasing (Khoshnoudi‐Nia et al., 2020). Essential oils are volatile oily liquids with strong and often pleasant odors obtained from different plant parts. Most essential oils are recognized as GRAS (Generally Recognized As Safe) by the Food and Drug Administration. Also, the European Union has approved their use as flavoring agents (Gutierrez et al., 2008).

On the other hand, climate change and global warming result in drought and a lack of water resources. Every year, millions of hectares of agricultural land lose their fertility due to soil salinity. Thus, efforts to find, develop, and use plant species resistant to salinity and water stress are particularly important (Firouzabadi et al., 2015). *Suaeda aegyptiaca* (their local name in Khuzestan is Googaleh and Kakol in Bushehr, Iran) is one of these plants. The literature review indicates the antioxidant and antimicrobial activity of *Suaeda* extract and essential oil. The bioactivity of the plant has been attributed to the presence of terpenoid compounds (α‐terpineol, camphor, borneol, etc.), phenolic, and flavonoid compounds (Askari et al., 2006; Choi et al., 2009; Li et al., 2020; Nayak et al., 2018). However, bioactive compounds are sensitive to environmental stresses and food processing conditions. All these challenges have limited the application of plant essential oils and extracts in the food industry. Encapsulation of bioactive compounds (entrapping one substance [active agent] into another substance [wall material] producing micro‐ or nanoparticles) will result in protecting these sensitive components from harsh environmental conditions, minimizing the sensory changes associated with strong aromas of extracts and essential oils, and enhancing the solubility of essential oil in aqueous systems (Khoshnoudi‐Nia et al., 2020, 2022).

Nanoemulsions are one of the most attractive and cost‐effective delivery systems based on nanotechnology produced by conventional processing methods in the food industry, and their properties can be easily manipulated and modified (McClements & Rao, 2011). Nanoemulsions are oil‐in‐water (O/W) or water‐in‐oil (W/O) emulsions with droplet size in the range of 20–200 nm with several unique properties including optical clarity, controlled release, suitable solubility, and bioactivity as well as physical stability (McClements & Rao, 2011; Villalobos‐Castillejos et al., 2018). Ultrasonication, high‐pressure homogenization and high shear homogenization are the most frequently techniques currently used to produce nanoemulsions (da Silva et al., 2022; McClements & Rao, 2011; Salvia‐Trujillo et al., 2015).

Emulsification by ultrasonic waves is considered as a cost‐effective and environmentally friendly method (Donsì & Ferrari, 2016). This method can produce emulsions with good physicochemical and thermodynamic stability, small droplet size and polydispersity index (PDI). In addition, the requirement of surfactant and energy in this technique is less compared to some other methods. The ultrasound waves create high‐energy cavitation which can break down large oil droplets into smaller ones (Nirmala et al., 2020; Pongsumpun et al., 2020).

Several studies have compared the bioactivity of some essential oils (such as laurel, tarragon, sage, ginger and lemon) and their nanoemulsions (Azizkhani et al., 2021; Moraes‐Lovison et al., 2017; Noori et al., 2018; Özogul et al., 2022; Yazgan, 2020; Yazgan et al., 2019). They have reported the promising potential of nanoemulsions as an effective treatment to improve the bioactivity of essential oils (Balasubramani et al., 2017; Hao et al., 2022; McClements et al., 2021; Yazgan, 2020). However, the bioactivity of *S. aegyptiaca* essential oil and its nanoemulsion have not been studied. Since this plant is resistant to salinity and water stress, investigating its bioactivity potential is important. This is the first study that deals with the antimicrobial and antioxidant properties of pure and emulsified *S. aegyptiaca* essential oil. Moreover, essential oils may exhibit variation and non‐producible bioactivity when evaluated in the laboratory and food matrix. Therefore, it is necessary to investigate the efficacy of essential oils in both conditions. Fish and seafood products are highly susceptible to microbial and chemical spoilage due to their high moisture, unsaturated lipid, and protein content (Khoshnoudi‐Nia & Moosavi‐Nasab, 2019a, 2019b). Thus, the bioactivity potential of Kakol essential oil and its nanoemulsion was evaluated on minced rainbow trout fish as a model food. Overall, the current study aims to identify the chemical composition of *S. aegyptiaca* essential oil by GC/MS; evaluate the physical properties of Kakol nanoemulsion; compare the antioxidant and antibacterial potential of the essential oil (pure and emulsified); and investigate the effect of *S. aegyptiaca* essential oil and its nanoemulsion on the shelf‐life of minced rainbow trout fish.

## 2 MATERIALS AND METHODS

### 2.1 Material

The aerial parts of Kakol (*S. aegyptiaca*) were collected from the Farashband region (Fars province, Iran); in March 2022. The aerial parts of Kakol were dried at shade (23 ± 3°C) until moisture content reach around 10% and kept in a dark bottle. All chemicals utilized in the current study were laboratory grade and purchased from Merck, Dr. Majalli, and Sigma‐Aldrich. The bacterial strain cultures (*Enterococcus faecalis* ATCC29212, *Klebsiella pneumoniae* ATCC700603, *Salmonella Paratyphi* A NCTC13, and *Staphylococcus aureus* ATCC 6538) were obtained from the *Iranian Research Organization for Science and Technology* (IROST).

### 2.2 Extraction of Kakol essential oil

The coarse dried powder of *S. aegyptiaca* (200 g) was transferred to a 3000‐mL Erlenmeyer flask containing distilled water (1500 mL). This Erlenmeyer flask was placed in an ultrasonic bath (V‐Clean1‐L6, Backer Co., 40 kHz, 150 W, 25°C) for 60 min. After this pretreatment, the pure *S. aegyptiaca* essential oil (PSAE) was extracted based on the hydro‐distillation technique for 5 h using a Clevenger apparatus (Azmiran). The essential oil was separated from distilled water three times by ethyl acetate and anhydrous sodium sulfate (Na_2_SO_4_) was used to dehydrate the essential oil. Then it was subjected to dryness in a Rotary evaporator under reduced pressure at room temperature. Kakol essential oil was stored in a dark glass bottle at 4 ± 1°C until analysis (Mohammed et al., 2019; Xing et al., 2019). The yield of the crude essential oil obtained by this method was 290 mg (0.29%).

### 2.3 Chemical composition of Nepeta essential oil

The volatile compound of Kakol essential oil was evaluated by a Gas Chromatography–Mass Spectrometry (GC/MS) system (Agilent 7890 A‐GC and Agilent 5975C‐MS; Agilent Technologies). Electron ionization was set at 70 eV and 150 μA (Agilent). Individual components of the essential oil were identified by comparison of their mass spectra with those in WILEY‐MS libraries or reported in the previous study (Salehi et al., 2007).

### 2.4 Nanoemulsion preparation

Oil‐in‐water nanoemulsion of *S. aegyptiaca* essential oil (NSAE) was prepared from a mixture of *S. aegyptiaca* (10% w/w), Tween 80 (2% w/w) and water (88% w/w). An ultrasonic homogenizer (Q500 Sonicator, Qsonica LLC; 500 W and 20 kHz) was applied to homogenize this mixture (15 min at 70 amplitudes). During this homogenization, the emulsion temperature was maintained at 15°C by ice around the beaker (Özogul et al., 2022).

### 2.5 Physical properties of nanoemulsion

The mean particle size and polydispersity index (PDI) of the emulsified droplets were measured by a dynamic light scattering (DLS) (DLS SZ‐100; Horiba) at 90° light scattering angle and 25°C (Kumar & Kumar, 2018). The viscosity of NSAE was measured by a rheometer (Stable Micro System, TA.XT2i). The stability of Kakol nanoemulsion was monitored for 2 weeks of storage at room temperature (around 25°C).

### 2.6 Free radical scavenging capacity (RSC)

The antioxidant activity of Kakol essential oil and its nanoemulsion were evaluated based on DPPH֯ (1,1‐diphenyl‐2‐picrylhydrazyl; absorbance at 515 nm) and ABTS (2, 2′‐azinobis (3‐ethylbenzothiazoline‐6‐sulfonic acid); absorbance at 734 nm) free radical scavenging capacity (RSC). the absorbance of the solutions was read by a Shimadzu 2501UV spectrophotometer. The IC_50_ value (50% inhibitory concentration), was evaluated using linear regression analysis of RSC values (Amira et al., 2012; Koleva et al., 2002).

### 2.7 Assignment of antibacterial activity

#### 2.7.1 Bacterial culture

The food‐related pathogens were used to evaluate the antimicrobial effect of the pure and emulsified Kakol essential oil as follows: *Enterococcus faecalis* ATCC29212, *Klebsiella pneumoniae* ATCC700603, *Salmonella Paratyphi* A NCTC13, and *Staphylococcus aureus* ATCC 6538.

#### 2.7.2 Bacterial inhibition assay

The bacterial inhibition effect of the *S. aegyptiaca* essential oil (pure and emulsified) was estimated based on the paper disc diffusion method (Murray et al., 1982) with slight modifications. The bacterial suspension (0.1 mL; 10^8^ CFU mL^−1^) was spread on the surface of the solid agar media in Petri dishes. Then, paper discs (diameter: 6 mm) were impregnated with 50 μL of Kakol essential oil or its nanoemulsion. Dried discs were placed over plates of nutrient agar seeded with each bacterium. Tetracycline (30 μg mL^−1^) and tween 80 were used as positive and negative controls, respectively. The plates were incubated at 37 ± 1°C for 24 h and inhibition zones around discs were estimated in millimeters (mm).

#### 2.7.3 Minimum inhibition/bactericidal concentration (MIC/MBC)

The MIC and MBC values of PSAE and NSAE against four food‐related pathogens were measured by broth dilution methods. For these analyses, Kakol essential oil (1 mL of pure or emulsified) was added to the first tube in each series and two‐fold diluted with sterile Muller Hinton Broth (MHB, Merck) to reach definitive concentration (50, 25, 12.5, 6.25, 3.12, 1.56, 0.78, 0.19 mg mL^−1^). Then, 1 mL of each bacterial suspension (10^6^ CFU mL^−1^) was added to each tube. The tubes were incubated at 37°C for 24 h under vigorous agitation. The tubes were analyzed for turbidity of the medium as an indicator for the growth of bacteria. The MIC values were considered as the lowest essential oil concentration inhibited visible growth of the tested microorganism. For the determination of MBC, the contents of tubes of MIC into Mueller Hinton Agar was subcultured at appropriate conditions for 24 h. The minimal concentration of the PSAE or NSAE showed no bacterial growth was defined as MBC (CLSI, 2008).

### 2.8 Effect of Kakol essential oil on shelf life of minced fish meat

#### 2.8.1 Fish samples preparation

Sixteen rainbow trout (*Oncorhynchus mykiss*) fishes (each one with an average weight of 550 ± 50 g) were freshly purchased from a local market (Shiraz, Iran) and immediately transferred to the laboratory in an ice box. After rigor mortis and removing internal organs, the fish samples were beheaded, filleted, washed with cold water, and finally minced (Parskhazar meat grinder, model MG‐1400R). Then the minced fish were divided into four groups with an approximate, which included: T_Co_: control sample (without essential oil); T_(PSAE)_: samples containing 1% w w^−1^ of PSAE; T_(NSAE)_: samples containing 1% w w^−1^ of NSAE, and T_(Tween 80)_: sample containing 1% Tween 80. The concentration of essential oil was selected based on previous studies (Araújo et al., 2018; da Silva et al., 2022; Hernández‐Hernández et al., 2017). The samples were packaged and encoded in sterile polyethylene bags and stored for 12 days at 4 ± 1°C. Chemical, microbial, and sensory experiments were done at 3‐day intervals on samples. But since all the samples obviously spoiled on the 12th day of storage (Stinky smell and slimy texture), the samples of this day were, therefore, discarded without testing.

#### 2.8.2 Thiobarbituric acid reactive substances (TBARS)

Thiobarbituric acid reactive substances values of minced fish samples were measured based on the colorimetric method (absorbance at 530 nm) and expressed by milligrams of malonaldehyde kg^−1^ sample (Namulema et al., 1999). The TBARS value of 1 mg MDA kg^−1^ was suggested as the threshold limit for the TBARS index (Khoshnoudi‐Nia & Moosavi‐Nasab, 2019a, 2019b).

#### 2.8.3 Total‐volatile basic nitrogen (TVB‐N)

Total‐volatile basic nitrogen content of samples was determined based on the steam distillation technique and by a Kjeldahl‐type apparatus (Kjeltec PDU‐500, PECO Co.). This index was reported in mg N 100 g^−1^ of the sample (Goulas & Kontominas, 2005). The acceptable limit for TVB‐N value was of 20 mg N 100 g^−1^ (Cheng & Sun, 2015; Khoshnoudi‐Nia & Moosavi‐Nasab, 2020).

#### 2.8.4 Microbial analysis

The total viable count (TVC) of samples was measured based on ISO 4833‐1:2013 method. Plates were incubated at 30°C for 72 h (ISO, 2013).

To evaluate the psychrotrophic microbial count (PMC), the incubation was done at 7°C for 10 days (ISO, 2019). The microbial load was recorded in terms of log colony‐forming units (CFUs) per gram (log_10_ CFU g^−1^). The maximum acceptable limit for TMC or PMC is considered 7 log CFU g^−1^ (ICMSF, 1986).

The Enumeration of Mesophilic Lactic Acid Bacteria was done based on plating on MRS agar medium at 30°C for 48 h (ISO, 1998).

The microbial load was recorded in terms of log colony‐forming units (CFUs) per gram (log_10_ CFU g^−1^). The maximum acceptable limit for TMC or PMC is considered 7 log CFU g^−1^ (ICMSF, 1986). While for LAB, the threshold limit was 6 log CFU g^−1^ (Jouki et al., 2014).

### 2.9 Statistical analysis

All analyses were conducted in three replicates and the results were reported as mean values and standard division (mean ± SD). Statistical analysis was performed based on ANOVA/General Linear Model (GLM). The significant differences between the means were tested by the Tukey test (*p* < .05). Statistical calculations were accomplished in SPSS software (IBM SPSS Statistics for Windows, Version 24.0, IBM).

## 3 RESULTS AND DISCUSSIONS

### 3.1 Chemical composition of the *S. aegyptiaca* essential oil

The Kakol (*S. aegyptiaca*) essential oil exhibited a pale‐yellow color and a pungent odor. The chemical composition of the essential oil was determined by GC/MS analysis (Table 1). The yield of this essential oil was 280 mg. Mohammed et al. (2019) also estimated the yield of *S. vermiculata* and *Salsola cyclophylla* essential oil to be 225 and 405 mg, respectively (Mohammed et al., 2019). Overall, 24 volatile compounds were identified in Kakol essential oil (82.84% of total volatiles). the major components of PSAE were oxygenated monoterpenes. Geranyl‐acetone (oxygenated monoterpene [ketone]: 30.52%), p‐Vinylguaiacol (Methoxyphenol: 10.66%), (e)‐β‐ionone (sesquiterpenes: 7.79%), 6,10,14‐trimethyl‐2‐pentadecanone (sesquiterpenes: 6.67%), (E)‐b‐Dmascenone (monoterpene ketone: 3.47%), benzyl methyl sulfide (benzene: 3.18%), n‐Nonanal (aldehyde/hydrocarbon: 3.14%), and n‐Decanal (aldehyde/hydrocarbon: 3.01%) were the main compounds of Kakol essential oil. Mohammed et al. (2019) identified 17 volatile compounds from *S. vermiculata* essential oil. They also reported that the oxygenated monoterpenes are major compounds of *S. vermiculata* essential oil which are known to have antimicrobial and antioxidant potential. The most important chemical compounds of *S. vermiculata* essential oil were ketones (ketone monoterpene: 28.98%; for example, camphor [28.74%], β‐damascenone [0.49%] and iso‐camphopinone [0.24%]) and alcohols (alcoholic monoterpene: 61.84%; e.g., borneol [33.77%] and α‐terpineol [22.78%]) (Mohammed et al., 2019).

**TABLE 1:** Chemical composition (%) of Kakol (*Suaeda aegyptiaca*) essential oil.

No.	Composition	Concentration (%)	Retention time (min)
1	(Z)‐2‐Octene	1.039	3.798
2	a‐Pinene	0.088	5.49
3	Benzaldehyde	0.489	6.18
4	Sabinene	0.488	6.53
5	1‐Octen‐3‐ol	0.761	6.63
6	6‐methyl‐5‐Hepten‐2‐one	1.433	6.87
7	n‐Octanal	0.205	7.19
8	Furfuryl methyl sulfide	0.329	7.25
9	Benzyl alcohol	0.592	8.42
10	Benzene acetaldehyde	0.982	8.74
11	n‐Octanol	0.802	9.56
12	Linalool	0.264	10.7
13	**n‐Nonanal**	**3.135**	**10.92**
14	(E)‐2‐Nonen‐1‐al	0.829	13.12
15	Benzyl methyl sulfide	3.175	13.44
16	Safranal	0.997	14.78
17	n‐Decanal	3.005	15.02
18	**p‐Vinylguaiacol**	**10.664**	**19.57**
19	**(E)‐b‐Dmascenone**	**3.465**	**22.52**
20	**Geranyl acetone**	**30.518**	**25.36**
21	**(E)‐β‐Ionone**	**7.789**	**26.65**
22	Butylated hydroxytoluene	2.618	27.71
23	Isopropyl tetradecanoate	2.498	39.26
24	**6,10,14‐trimethyl‐2‐Pentadecanone**	**6.673**	**39.772**

*Note*: The bold rows identified the main compounds of Kakol essential oil.

### 3.2 Physical properties of the nanoemulsion

**FIGURE 1:**
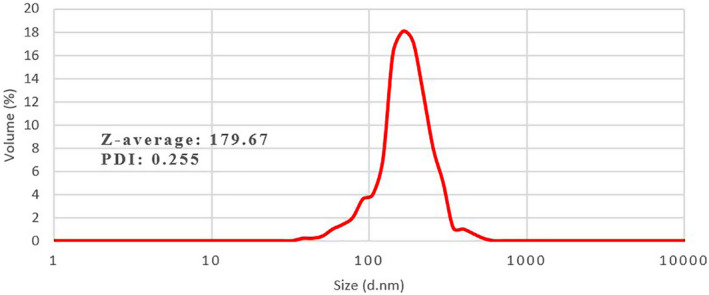
The particle size distribution of the Kakol (*Suaeda aegyptiaca*) essential oil nanoemulsion.

Mean droplet diameter and PDI of NSAE were found 179.67 and 0.255 nm, respectively (Figure 1). A nanodispersion with a small PDI value (PDI < 0.3) indicates a relatively narrow distribution size and consequently stability and homogeneity of the essential oil in an aqueous system. Increasing PDI may lead to the Ostwald ripening phenomenon (McClements et al., 2021). These values were comparable with those reported for the nanoemulsions prepared similarly. Özogul et al. (2022) estimated the particle size and PDI of 247.52 and 0.336 nm for nanoemulsion based on laurel essential oil (Özogul et al., 2022). Lee et al. (2019) reported larger particles (221.3 nm) with a narrower particle size distribution (PDI = 0. 251) for oregano nanoemulsion; while the mean diameter and PDI of macroemulsion of oregano essential oil were 1420.47 ± 111 and 0.709 ± 0.04, respectively. These authors have pointed to the role of the high energy of ultrasound waves to break bigger particles down into smaller ones and improve homogeneity (Lee et al., 2019). The physical properties of a nanoemulsion can be affected by its formulation (type and concentration of surfactant, essential oil, and aqueous phase) and preparation conditions (shear forces and turbulence produced by the ultrasonic homogenizer) (Özogul et al., 2022; Pandey et al., 2022).

The viscosity of a nanoemulsion can affect the physicochemical properties of a nanoemulsion and determines the type of emulsion which oil‐in‐water emulsions show a lower viscosity than water‐in‐oil emulsions (Yazgan, 2020). Viscosity can also be considered as a function of particle diameter. The viscosity of the NSAE was 0.96 cP which was comparable to the viscosity of lemon essential oil nanoemulsion (cp 0.88) (Salehi, et al., 2007; Yazgan et al., 2019). Yazgan et al. (2019) reported that the viscosity of lemon essential oil was about 5 cp (McClements & Rao, 2011). Thus, incorporating essential oil into nanoemulsion greatly reduces the viscosity. The viscosity of sage (1.51 cp) and laurel (1.41 cp) nanoemulsion was slightly higher than those reported in the current study (Özogul et al., 2022; Yazgan, 2020). This difference can be related to various nanoemulsion formulations. Also, NSAE showed acceptable stability during 2 weeks of storage at room temperature.

### 3.3 Antioxidant activity

**FIGURE 2:**
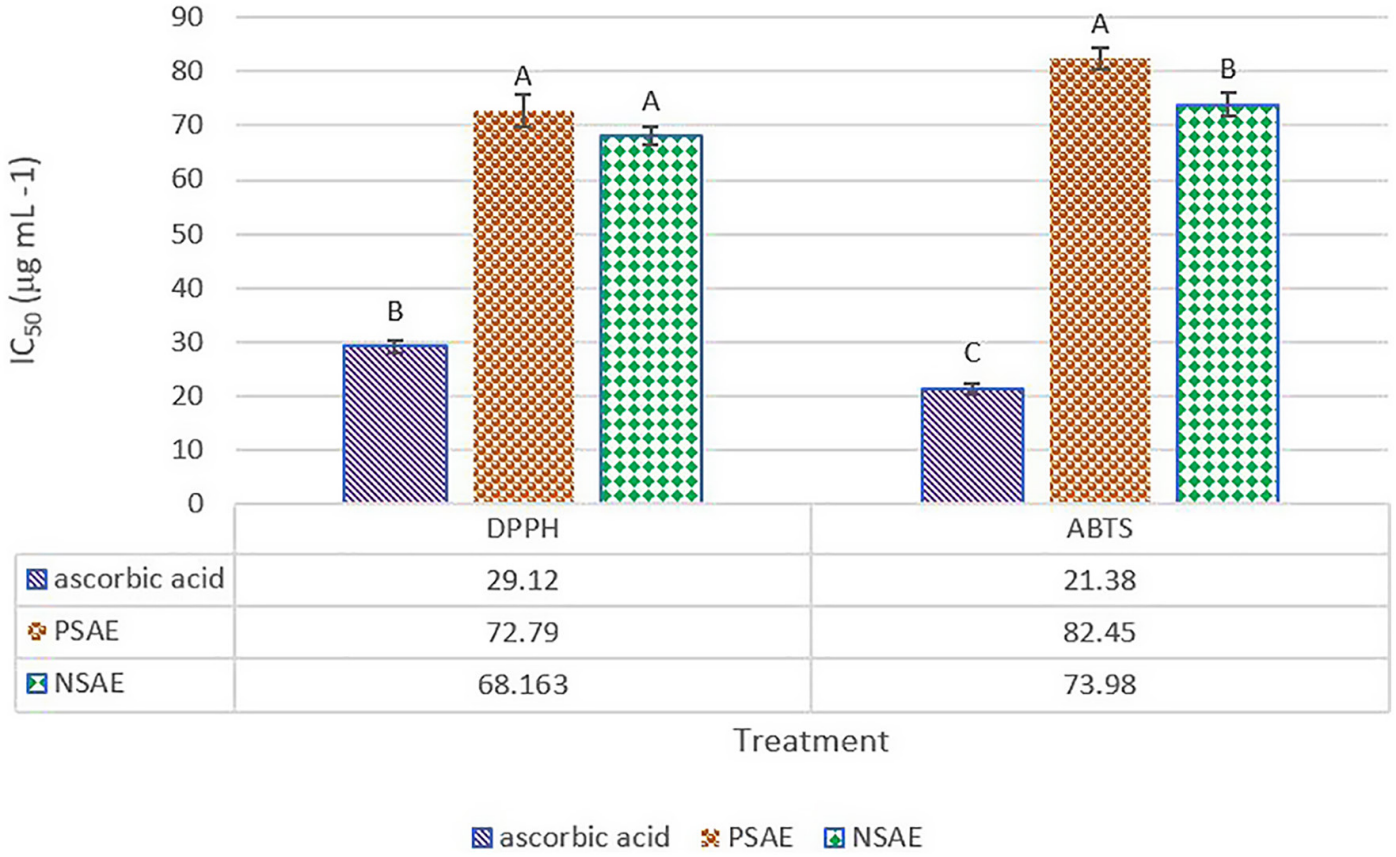
The antioxidant potential (based on DPPH and ABTS^+^ free radical scavenging activities) of pure Kakol essential oil (PSAE) and its nanoemulsion (NSAE) as compared to ascorbic acid. Different capital letter for each factor indicated significant differences between various treatment (*p* < .05).

The antioxidant potential (based on DPPH and ABTS^+^ free radical scavenging activities) of Kakol essential oil and its nanoemulsion as compared to ascorbic acid was shown in Figure 2. lower IC_50_ values indicate a higher antioxidant capacity. Antioxidant activity of a compound can be classified as inactive (IC_50_ > 250 μg mL^−1^); weak (100 < IC_50_ < 250 μg mL^−1^), moderate (50 < IC_50_ < 250 μg mL^−1^), strong (10 < IC_50_ < 50 μg mL^−1^), and very strong (IC_50_ < 10 μg mL^−1^) (Phongpaichit et al., 2007). As presented in Figure 2, Kakol essential oil (pure or nanoemulsified) showed a moderate antioxidant potential (IC_50 (DPPH)_: PSAE = 72.79 and PSAE = 68.16 μg mL^−1^; IC_50 (ABTS)_: PSAE = 82.45 and NSAE = 73.98 μg mL^−1^). The findings were comparable to those reported for *Suaeda maritima* essential oil (IC_50 (DPPH)_ = 53.53 μg mL^−1^) (Nayak et al., 2018). The antioxidant activity of *S. aegyptiaca* essential oil can be attributed to the terpenes, especially the oxygenated monoterpenes and phenolic terpenoids (Al‐Kanaany, 2020). However, the antioxidant activity of the PSAE and NSAE were lower than standard ascorbic acid (AA) (based on DPPH: PSAE = 40.02 ± 0.83% and NSAE = 42.73 ± 1.62% of AA and based on ABTS: PSAE = 25.96 ± 1.81% and NSAE = 28.93 ± 1.90% of AA).

Although the ABTS index allows the measurement of hydrophilic and hydrophobic antioxidants, this index is more sensitive for hydrophilic compounds (Floegel et al., 2011). Probably, for this reason, the IC_50_ level recorded for ABTS of PSAE and NSAE, with a hydrophobic base, is higher than the IC_50_ level required to scavenge the DPPH‐free radical.

Although there was no significant difference between the IC_50(DPPH)_ of the Kakol essential oil and its nanoemulsion, the IC_50(ABTS)_ of NSAE was significantly lower than PSAE (*p* < .05). It can be related to the hydrophilic base of NSAE (Floegel et al., 2011). By reducing the particle size of essential oil during nanoemulsion processing, surface area to volume increases. It leads to increasing the bioactivity of essential oil and enhancing the interaction between bioactive compounds and free radicals. Thus, a certain bioactivity (e.g., antioxidant activity) can be exhibited in a lower concentration of essential oil as compared to a pure essential oil (Balasubramani et al., 2017; Seibert et al., 2019; Sundararajan et al., 2018).

### 3.4 Antimicrobial activity

Table 2 shows the antibacterial properties of the PSAE and NSAE against Gram‐positive (*E. faecalis* and *S. aureus*) and Gram‐negative (*S. paratyphi* and *K. pneumonia*) bacteria. *E. faecalis* and *K. pneumonia* were the most sensitive and resistant bacteria to the essential oil (19.72 and 10.53 mm, respectively). The most sensitive bacteria to the nanoemulsion was *S. aureus* (18.41 mm) and *K. pneumonia* was resistant to NSAE. There was no significant difference between the antibacterial effect of Kakol essential oil and its nanoemulsion (except *K. pneumonia*). The antibacterial potential of PSAE against the Gram‐positive bacteria was superior to NSAE, while for *S. paratyphi*, the performance of NSAE was better than PSAE (*p* < .05). A higher local concentration of bioactive compounds in NSAE could be a reason for the suitable activity of NSAE in a lower concentration of essential oil as compared to PSAE (Garzoli et al., 2020).

**TABLE 2:** Antimicrobial activity of Kakol (*Suaeda aegyptiaca*) essential oil and its nanoemulsion against foodborne pathogen bacteria by disc diffusion method.

Food‐borne pathogen bacteria	Disc‐diffusion method (mm)			Ratio to tetracycline	
	PSAE	NSAE	Tetracycline	PSAE	NSAE
*Enterococcus faecalis*	19.72 ± 0.72^B^	17.75 ± 0.61^B^	30.67 ± 2.19^A^	64.46 ± 2.49	58.00 ± 2.28
*Staphylococcus aureus*	18.95 ± 0.97^B^	18.07 ± 0.12^B^	24.57 ± 0.75^A^	77.18 ± 4.95	73.59 ± 2.66
*Salmonella Paratyphi* A	12.63 ± 0.67^B^	14.20 ± 0.59^B^	22.44 ± 0.87^A^	56.28 ± 1.05	63.27 ± 0.78
*Klebsiella pneumoniae*	10.53 ± 1.02^B^	R	28.70 ± 1.44^A^	36.75 ± 3.82	‐

*Note*: R: resistant, value represents mean ± SD. Different superscripts (A, B) in the same column correspond to a significant difference (*p* < .05). Tween 80 used as a negative control, did not show any inhibition zones against four food‐related pathogens.

Abbreviations: NSAE, nano‐emulsified Kakol essential oil; PSAE, pure Kakol (*Suaeda aegyptiaca*) essential oil.

Numerous bioactive compounds with diverse chemical groups are found in essential oils, so the effect mechanism of each antimicrobial compound can be different. Phenols, terpenes, terpenoids, and other aromatic and aliphatic compounds can interact with the bacterial phospholipid cell membrane, penetrate the bacterial cell by increasing membrane permeability, and leak out the cytoplasmic content. Thus, the pH of the cell changes, which can lead to enzyme denaturation, changes in cell morphology and finally death of the bacterial cell (Di Pasqua et al., 2007; Seow et al., 2014; Youseftabar‐Miri et al., 2021).

As seen in Table 2, Gram‐negative bacteria, especially *K. pneumonia*, were more resistant to PSAE and NSAE as compared to Gram‐positive bacteria. The lipopolysaccharide (LPS) membrane of Gram‐negative bacteria protects the cytoplasmic membrane from the antimicrobial action of essential oil (Nazzaro et al., 2013; Youseftabar‐Miri et al., 2021).

Comparison performance of essential oil and nanoemulsion with tetracycline showed that tetracycline exhibited stronger antimicrobial activity against all food‐borne pathogen bacteria tested. However, the antibacterial performance of Kakol essential oil nanoemulsion (except *K. pneumonia*) was acceptable (PSAE: 56.28%–77.18% and NSAE: 58.00–73.59).

### 3.5 Determination of minimum inhibition/bactericidal concentration (MIC and MBC)

The minimum inhibition and bactericidal concentrations of *S. aegyptiaca* essential oil (pure and nanoemulsified) on four food‐related pathogens are shown in Table 3. The MIC values of PSAE against the tested bacteria ranged from 3.13 mg mL^−1^ to >50.00 mg mL^−1^. These values were between 6.25 and >50 mg mL^−1^ for NASE. MBC values of PSAE and NSAE ranged from 12.5 to >50 mg mL^−1^. The highest antimicrobial activity of PSAE was against *E. faecalis* (MIC = 3.13 and MBC = 12.5 mg mL^−1^). The MIC of PSAE and NSAE for both Gram‐positive bacteria was 6.25 mg mL^−1^, while the MBC for *E. faecalis* and *S. aureus* bacteria was 12.5 and 25 mg mL^−1^, respectively. As shown in Section 3.4, the antibacterial potential of Kakol essential oil (pure and emulsified) against Gram‐negative bacteria was weaker than Gram‐positive. MIC and MBC for *S. Paratyphi* A were 25 and 50 mg mL^−1^, respectively; while these values for *Klebsiella pneumoniae* were more than 50 mg mL^−1^. Özogul et al. (2022) found that the MIC values of laurel essential oil against *S. aureus* ATCC 29.21, *E. faecalis* ATCC 29.212, *S. paratyphi* A NCTC13, and *K. pneumoniae* ATCC 700.603 to be 12.5, 12.5, 3.13, and 12.5 mg mL^−1^, respectively. The MBC values for these four strains were >25 mg mL^−1^ (except for *S. paratyphi*, which was 12.5 mg mL^−1^) (Özogul et al., 2022). Therefore, the antibacterial potential of Kakol essential oil against Gram‐positive was slightly better than those reported for laurel essential. However, Kakol essential oil showed less effectiveness against Gram‐negative bacteria than laurel essential oil.

**TABLE 3:** MIC and MBC determination of Kakol (*Suaeda aegyptiaca*) essential oil and its nanoemulsion on food‐related pathogens.

Food‐borne pathogen bacteria	MIC (mg mL^−1^)		MBC (mg mL^−1^)	
	PSAE	PSAE	PSAE	NSAE
*Enterococcus faecalis*	3.13	6.25	12.5	12.5
*Staphylococcus aureus*	6.25	6.25	12.5	25
*Salmonella Paratyphi* A	25	25	>50	50
*Klebsiella pneumoniae*	>50	>50	>50	>50

Abbreviations: MBC, minimum bactericidal concentration; MIC, minimum inhibition concentration; NSAE, nanoemulsified Kakol essential oil; PSAE, pure Kakol (*Suaeda aegyptiaca*) essential oil.

Monoterpene compounds of essential oils have significant antimicrobial activity due to their lipophilic properties that lead to cell membrane disruption. Moreover, changes in the phospholipids structure of the membrane results in energy loss, changes in DNA and RNA synthesis, and disruption of protein transport (Yazgan et al., 2019). Terpenic oils in nanoscale systems provide several innovative and biological properties such as biodegradability, higher permeability, thermal stability, solubility, rigidity, and crystallinity (Almadiy et al., 2016). Therefore, they show acceptable bioactivity in a lower concentration than pure essential oil. The antibacterial activity of hydrocarbons, sesquiterpenes, and oxygenated monoterpenes of various essential oils has been proven by previous studies (Chuesiang et al., 2019; Lee et al., 2019; Noori et al., 2018).

### 3.6 Shelf‐life of rainbow trout

#### 3.6.1 Thiobarbituric acid reactive substance (TBARS)

**FIGURE 3:**
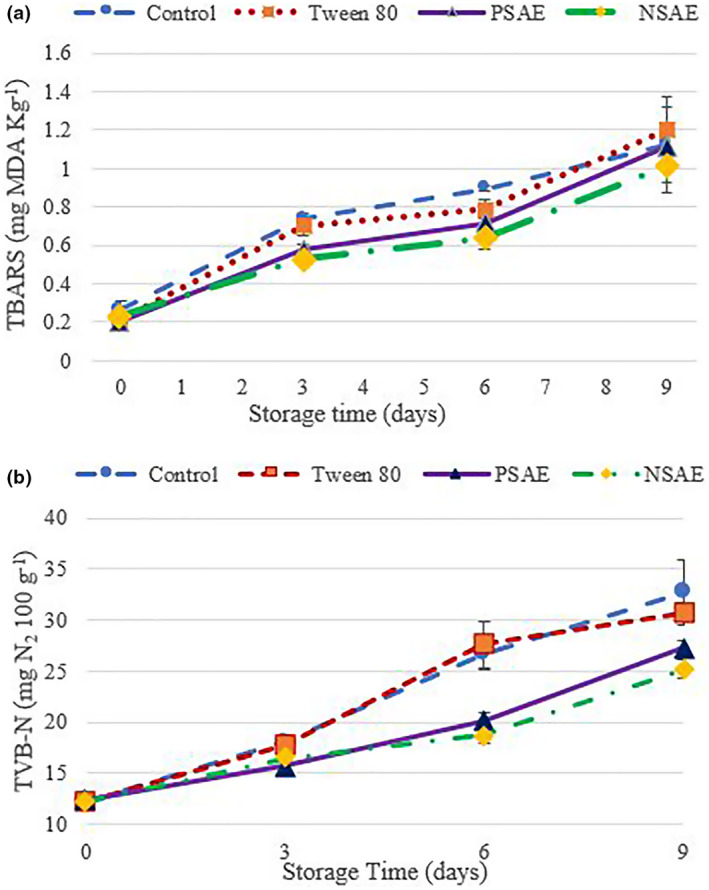
The effect of Kakol (*Suaeda aegyptiaca*) essential oil (PSAE) and its nanoemulsion (NSAE) on (a) thiobarbituric acid reactive substance (TBARS) and (b) total volatile basic‐nitrogen (TVB‐N) values of minced rainbow trout fish during storage at 4 ± 1°C. Control: Minced fish sample without essential oil; PSAE: Fish samples containing 1% w w^−1^ of pure *S. aegyptiaca* essential oil; NSAE: Fish samples containing 1% w w^−1^ of nanoemulsified *S. aegyptiaca* essential oil and Tween_80_: Fish samples containing 1% Tween 80.

Fish and other seafood generally contain high levels of polyunsaturated fatty acids, the oxidation of these sensitive lipids leads to undesirable changes in the color and taste (rancidity) of fish, as well as the accumulation of toxic compounds, which ultimately results in a decrease in fish quality and shelf‐life (Das et al., 2020; Khoshnoudi‐Nia & Moosavi‐Nasab, 2019a, 2019b). The effect of Kakol essential oil and its nanoemulsion on the TBARS index of minced rainbow trout samples during 9 days of storage at 4 ± 1°C is shown in Figure 3. The initial values of TBARS was around 0.24 mg MDA kg^−1^, which during storage reached higher than 1 mg MDA kg^−1^ (threshold limit) in all samples (*p* < .05). Although the difference between various samples was not significant, the TBARS values of samples treated with the essential oil (pure or emulsified) were lower than other ones. On the last day of storage, the TBARS value of T_(PSAE)_ was almost equal to the control sample, but the difference between the control sample and T_(NSAE)_ was greater (*p* > .05). The encapsulation of essential oil in nanoemulsion can protect the bioactive compounds of Kakol essential oil so NSAE showed similar antioxidant activity at a lower concentration of essential oil than PSAE.

The lack of significant differences between samples can be related to the fluctuations of the TBARS value and the reaction of malondialdehyde with fish compounds. Several authors reported that the investigation of TBARS value is not adequate to evaluate the quality and shelf‐life of fish due to these fluctuations (Jouki et al., 2014; Khoshnoudi‐Nia & Moosavi‐Nasab, 2019a, 2019b). On the 9th day of storage, the TBARS value of T_(NSAE)_ was still considered within the acceptable limit. However, the TBARS values of other samples were beyond 1 mg MDA kg^−1^. Application of the essential oil or nanoemulsion in the form of edible coatings may reduce the interaction of bioactive compounds and with food compositions and induces longer bioactivity (Ghani et al., 2018; Moraes‐Lovison et al., 2017).

#### 3.6.2 Total volatile basic nitrogen (TVB‐N)

The investigation of the main effect of essential oil treatment on the TVB‐N contents of minced fish showed that the lowest TVB‐N value was obtained for T_(NSAE)_ (17.80 mg N_2_ 100 g^−1^) and T_(PSAE)_ (18.76 mg N_2_ 100 g^−1^). Although the difference between the two samples was not significant, the difference between them with T_Co_ (22.34 mg N_2_ 100 g^−1^) and T_(Tween 80)_ (21.88 mg N_2_ 100 g^−1^) was significant. Over time, the TVB‐N content increased due to microbial spoilage and the destruction of fish tissue. Unlike the TBARS index, the interaction effect of treatment (essential oil) and time on TVB‐N was also significant (*p* < .05). Figure 3b showed the effect of essential oil on TVB‐N during storage time. Until the 3rd day of storage, there was no significant difference between samples. On the 6th day, T_Co_ and T_(Tween 80)_ exceeded the standard limit (20 mg N_2_ 100 g^−1^ TVB‐N), while the other two samples were still within the acceptable limit and there was a significant difference between these two groups (*p* < .05). On the last day of storage, although the TVB‐N values of all samples were >20 mg N_2_ 100 g^−1^, the highest TVB‐N content was recorded for control one (*p* < .05). The difference between T_(Tween 80)_ with T_Co_ and T_(PSAE)_ was not significant (*p* > .05). Furthermore, there was no significant difference between T_(PSAE)_ and T_(NSAE)_.

Total volatile basic nitrogen is a combination of trimethylamine (TMA), dimethylamine (DMA), ammonia, and other volatile nitrogen compounds. DMA is generally produced by endogenous enzymes and TMA is generated by bacterial enzymes; Therefore, the results of TVB‐N content generally has a relationship with the microbial load of the product (Moosavi‐Nasab et al., 2021; Rong et al., 2009). Since on an industrial scale, the measurement of the microbial load is a time‐consuming process, the TVB‐N value is introduced by regulatory authorities in some countries as a mandatory parameter to assess the microbial quality of meat products (Moosavi‐Nasab et al., 2021). Therefore, the effect of Kakol essential oil (pure and emulsified) can be related to its antimicrobial properties (Fan et al., 2008). Durmuş et al. (2020) reported that nanoemulsion of citrus essential oils significantly delay the increasing trend of TVB‐N value in rainbow trout.

Although in the first days of storage, the TVB‐N level of T_(PSAE)_ was lower than T_(NSAE)_, over time, this difference disappeared, and finally, on the last day, the performance of NSAE was superior to PSAE to control TVB‐N content.

The lower TVB‐N level of T_(PSAE)_ in the first days can be attributed to a higher concentration of essential oil. However, a free essential oil is more volatile and can be diluted in fish oil, while the encapsulation of the essential oil protect bio compounds (da Silva et al., 2022). These results were in line with the findings of previous studies about the effectiveness of essential oil and nanoemulsions in controlling the TVB‐N formation in meat products (Abdou et al., 2018; Pouryousef et al., 2022; Wang et al., 2022).

#### 3.6.3 Total viable count (TVC)

**FIGURE 4:**
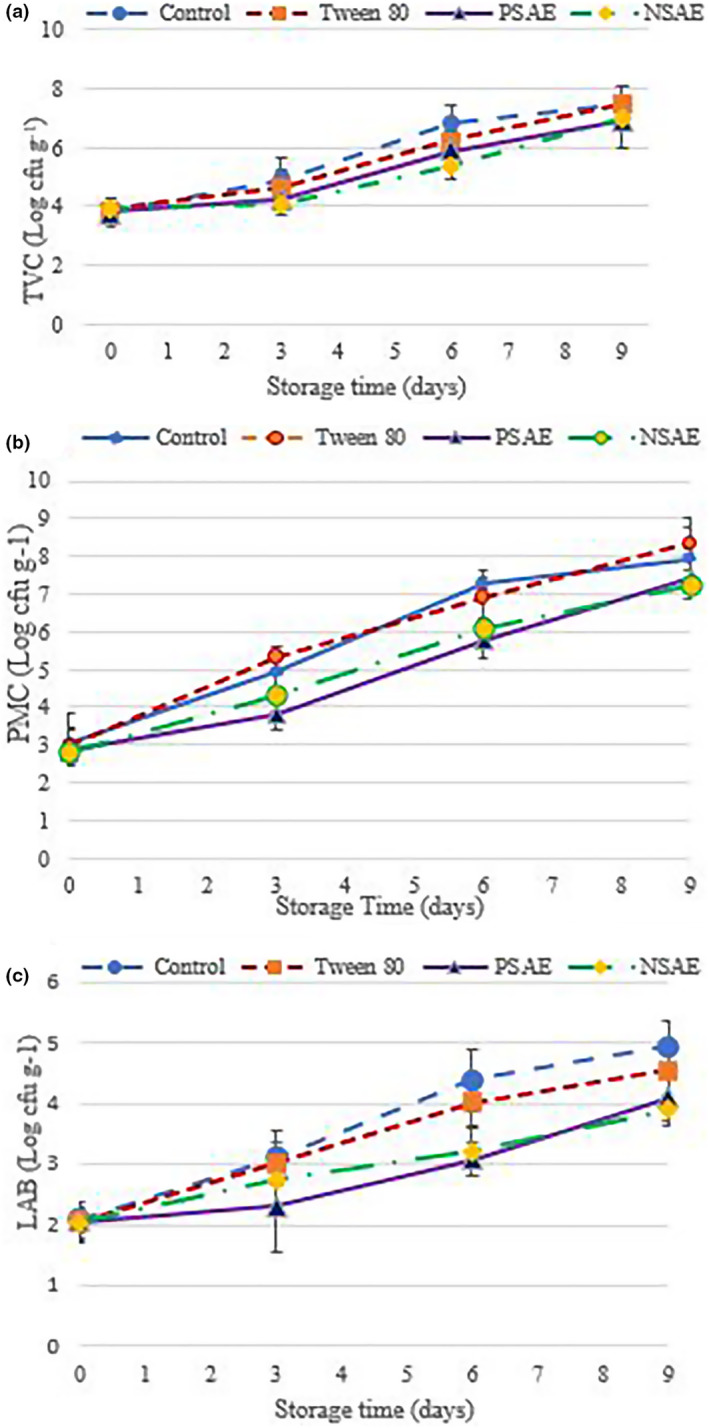
The effect of Kakol (*Suaeda aegyptiaca*) essential oil (PSAE) and its nanoemulsion (NSAE) on (a) total viable count (TVC); (b) psychrophilic microbial counts (PMC); and (c) mesophilic Lactic acid bacteria (LAB) of minced rainbow trout fish during storage at 4 ± 1°C. Control: Minced fish sample without essential oil; PSAE: Fish samples containing 1% w w^−1^ of pure *S. aegyptiaca* essential oil; NSAE: Fish samples containing 1% w w^−1^ of nanoemulsified *S. aegyptiaca* essential oil and Tween_80_: Fish samples containing 1% Tween 80.

As shown in Figure 4a, on the first day of storage, the TVC of samples was between 3.79 and 3.91 Log CFU g^−1^. TVC value between 2 and 4 Log cfu g^−1^ indicates the acceptable quality of fish meat (Jouki et al., 2014; Khoshnoudi‐Nia et al., 2018; Khoshnoudi‐Nia & Moosavi‐Nasab, 2019a, 2019b; Mohan et al., 2018; Shokri et al., 2015). This microbial load may be due to the contamination that occurred during processing activities (e.g., filleting, gutting, mincing, and packaging of fish) (Ahmad et al., 2012). During 9 days of storage, the TVC reached 6.88 Log cfu g^−1^ for T_(PSAE)_ to 7.51 Log cfu g^−1^ for T_(Tween 80)_ and there was an increasing trend in all samples.

The comparison of the main effect of essential oil treatment on the microbial load of samples showed that the TVC of T_(NSAE)_ and T(_PSAE_) was significantly lower than T_Co_. This can be related to the antimicrobial potential of Kakol essential oil and its nanoemulsion (see Section 3.4). The results were in agreement with the findings of previous studies about the effect of essential oils and their nanoemulsions on the microbial load of meat products (Abdou et al., 2018; Durmuş et al., 2020; Noori et al., 2018; Pouryousef et al., 2022). However, there was no significant effect between T_(PSAE)_ with T_(NSAE)_ and T_(Tween80)_ at the 5% level. Also, the interaction effect of time and treatment was not significant (*p* > .05). However, the sample containing essential oil showed a lower TVC during storage. Although the difference between T_(NSAE)_ and T(_PSAE_) was not significant, nanoemulsion showed a similar effect at a lower concentration of essential oil. Since the yield of essential oils is very low, it can be considered from an economic point of view. Based on this parameter, while T_(NSAE)_ was within the threshold limit (7 Log CFU g^−1^) for 9 days, T_Co_ and T_(Tween 80)_ showed acceptable quality for around 7 days, this time for T_(PSAE)_ was 8 days.

#### 3.6.4 Psychrophilic microbial counts (PMC)

Aerobic and Psychrophilic Gram‐negative (e.g., *Pseudomonas*, *Alteromonas*, *Moraxella*, *Shewanella*, and *Flavobacterium*) were dominant in the spoiled fish and seafood products stored at refrigerated temperatures (Fraser & Sumar, 1998; Gennari et al., 1999; Hou et al., 2013). The effect of Kakol essential oil on the PMC values of minced rainbow trout during storage is exhibits in Figure 4b. On the first day of storage, the PMC values of samples were around 3 Log cfu g^−1^. After 3 days of storage, this value increased significantly in all samples except T_(PSAE)_. On the 6th day of storage, the PMC value of the control sample reached to threshold point (7 Log cfu g^−1^). At this time, the lowest PMC was recorded for T_(PSAE)_. The difference between the samples containing essential oil and T_(Tween 80)_ was not significant (*p* > .05). But there is a significant difference between these samples and the control one. On the 9th day of storage, the highest and lowest PMC was for T_(Tween 80)_ and T_(NSAE)_, respectively (*p* > .05). The PMC level of all samples exceeded the acceptable limit.

Although the interaction effect of time and treatment was not significant at the 5% level, the investigation of the main effect of the essential oil treatment showed that the PMC of T_(PSAE)_ (4.96 Log cfu g^−1^) and T_(NSAE)_ (5.1 Log cfu g^−1^) was significantly lower than T_Co_ (5.78 Log cfu g^−1^) and T_(Tween80)_ (5.89 Log cfu g^−1^). The effectiveness of essential oils and their nanoemulsions for controlling PMC of fish meat was reported by several previous authors (Durmuş et al., 2020; Hassanzadazar et al., 2021). Although the essential oil showed relatively good microbial potential in the plate medium, these compounds reduced the PMC in samples by less than 1 Log cfu g^−1^. It can be related to the diluting of the essential oil in the fish oil and the reaction of its bioactive compounds with the fish compositions. On the other hand, PMCs are generally Gram‐negative bacteria that have relatively low sensitivity to the essential oil and its nanoemulsion due to the presence of a protective LPS on the bacterial cell wall (da Silva et al., 2022).

#### 3.6.5 Mesophilic lactic acid bacteria (LAB)

As shown in Figure 4c, the initial LAB content of samples was around 2 Log cfu g^−1^, and on the 3rd day of storage, this value reached around 3.03–2.3 Log cfu g^−1^ (*p* > .05). After 6 days of storage, no significant difference was observed in the LAB of samples containing Kakol essential oil. On this day, only the difference between the T_(PSAE)_ and T_Co_ was significant (*p* < .05). On the last day, despite a difference of around 1 Log cfu g^−1^ between T_(NSAE)_ and T_Co_ (3.91 vs. 4.94 Log cfu g^−1^), this difference was not significant. At this time, the antibacterial activity of NSAE was more than PSAE (*p* > .05).

Regarding the main effect of essential oil treatment, the difference between T_Co_ and the samples containing essential oil (T_(PSAE)_: 2.89 Log cfu g^−1^ and T_(NSAE)_: 2.97 Log cfu g^−1^) was significant. The difference between the control sample (3.64 Log cfu g^−1^) and T_(Tween 80)_ (3.42 Log cfu g^−1^) was not significant. Ahmad et al. (2012) reported that when 0.4% of cumin essential oil was added to the culture medium of lactic acid bacteria, 4 Log cfu g^−1^ of reduction was achieved. However, in model food (fish sample) this reduction was much lower due to the protective effect of food components on bacteria (Ahmad et al., 2012). Higher concentrations of essential oil and nanoemulsion may improve their effectiveness. Despite the obvious spoilage signs, based on the threshold limit of LAB (6 Log cfu g^−1^), all samples were acceptable. The optimum temperature for the growth of lactic acid bacteria was around 37°C, so the bacterial growth is slow at refrigerator temperature (Wen et al., 2022). Therefore, this index cannot be a suitable parameter for estimating the shelf life of fish storage. These results were in line with those reported by previous studies (Dehghani et al., 2018; Jouki et al., 2014; Khalili et al., 2021; Khoshnoudi‐Nia et al., 2022).

## 4 CONCLUSION

The current study investigated the antiradical and antibacterial potential of pure Kakol (*S. aegyptiaca*) essential oil (PSAE) and its nanoemulsion (NSAE) in both laboratory medium and food matrix (minced rainbow trout fish). Kakol essential oil and its nanoemulsion showed moderate antiradical power against DPPH and ABTS free radicals. The antibacterial potential of Kakol essential oil (pure and emulsified) against Gram‐positive was higher than Gram‐negative. The addition of PSAE and NSAE to minced rainbow trout resulted in enhancing the shelf‐life of fish between 12.86% (based on the TBARS index) and 60% (based on the PMC index) compared to the control sample. Although the conversion of essential oil into nanoemulsion had no significant effect on the shelf‐life of minced fish, using nanoemulsion greatly reduced the level of essential oil consumption. Since the yield of essential oils is low, it justifies the use of nanoemulsions in the food industry. On the other hand, the cost of surfactants and nanoemulsion production technology should be considered in making a decision on which technology to use. Further study is required to clarify the gaps related to particle size standardization, the synergistic effect between essential oils and the stability of nanoemulsion. In addition, more research is needed to optimize the concentration of PSAE and NSAE in meat products and evaluate the reaction of bioactive compounds of essential oils with the chemical compounds of meat. By developing research on using essential oils (pure and emulsified), it will be possible to identify standardized rules for the application of essential oils in food as natural preservatives.

## AUTHOR CONTRIBUTIONS

**Payam Zibaee:** Conceptualization (supporting); data curation (supporting); methodology (supporting); writing – original draft (supporting). **Mohammad‐Amin Shamekhi:** Formal analysis (equal); funding acquisition (equal); resources (equal); supervision (equal); validation (equal); visualization (equal); writing – original draft (equal).

## FUNDING INFORMATION

The authors did not receive support from any organization for the submitted work. This study was conducted at the personal expense of the authors and had no sponsorship.

## CONFLICT OF INTEREST STATEMENT

The authors declare that they have no conflict of interest.

## ETHICS STATEMENT

This article does not contain any studies with human participants or animals performed by any of the authors.

## Data Availability

The datasets generated and/or analyzed during the current study are available from the corresponding author on reasonable request.
